# Pathophysiology and probable etiology of cerebral small vessel disease in vascular dementia and Alzheimer’s disease

**DOI:** 10.1186/s13024-023-00640-5

**Published:** 2023-07-11

**Authors:** Yasuteru Inoue, Francis Shue, Guojun Bu, Takahisa Kanekiyo

**Affiliations:** 1 Department of Neuroscience, Mayo Clinic, 4500 San Pablo Road, Jacksonville, FL 32224 USA; 2 SciNeuro Pharmaceuticals, Rockville, MD 20850 USA

**Keywords:** Vascular cognitive impairment and dementia (VCID), Cerebral small vessel disease (cSVD), Blood–brain barriers (BBB), Glymphatic drainage, Intramural periarterial drainage (IPAD), Arteriolosclerosis, Cerebral amyloid angiopathy (CAA), Hypoperfusion/Hypoxia, Vascular inflammation

## Abstract

Vascular cognitive impairment and dementia (VCID) is commonly caused by vascular injuries in cerebral large and small vessels and is a key driver of age-related cognitive decline. Severe VCID includes post-stroke dementia, subcortical ischemic vascular dementia, multi-infarct dementia, and mixed dementia. While VCID is acknowledged as the second most common form of dementia after Alzheimer’s disease (AD) accounting for 20% of dementia cases, VCID and AD frequently coexist. In VCID, cerebral small vessel disease (cSVD) often affects arterioles, capillaries, and venules, where arteriolosclerosis and cerebral amyloid angiopathy (CAA) are major pathologies. White matter hyperintensities, recent small subcortical infarcts, lacunes of presumed vascular origin, enlarged perivascular space, microbleeds, and brain atrophy are neuroimaging hallmarks of cSVD. The current primary approach to cSVD treatment is to control vascular risk factors such as hypertension, dyslipidemia, diabetes, and smoking. However, causal therapeutic strategies have not been established partly due to the heterogeneous pathogenesis of cSVD. In this review, we summarize the pathophysiology of cSVD and discuss the probable etiological pathways by focusing on hypoperfusion/hypoxia, blood–brain barriers (BBB) dysregulation, brain fluid drainage disturbances, and vascular inflammation to define potential diagnostic and therapeutic targets for cSVD.

## Background

Vascular cognitive impairment and dementia (VCID) is caused by various types of cerebrovascular damage such as microvascular dysfunction and large vessel stroke, impacting a large percentage of the world’s population as society ages [1]. Epidemiological studies have demonstrated that VCID is the second most common form of dementia after Alzheimer’s disease (AD), accounting for approximately 20% of dementia cases [2]. Although the clinical diagnostic criteria are somewhat vague, VCID is characterized by cognitive decline through neuropsychological testing and detection of cerebrovascular lesions through neuroimaging or clinical stroke history [3].

**Fig. 1:**
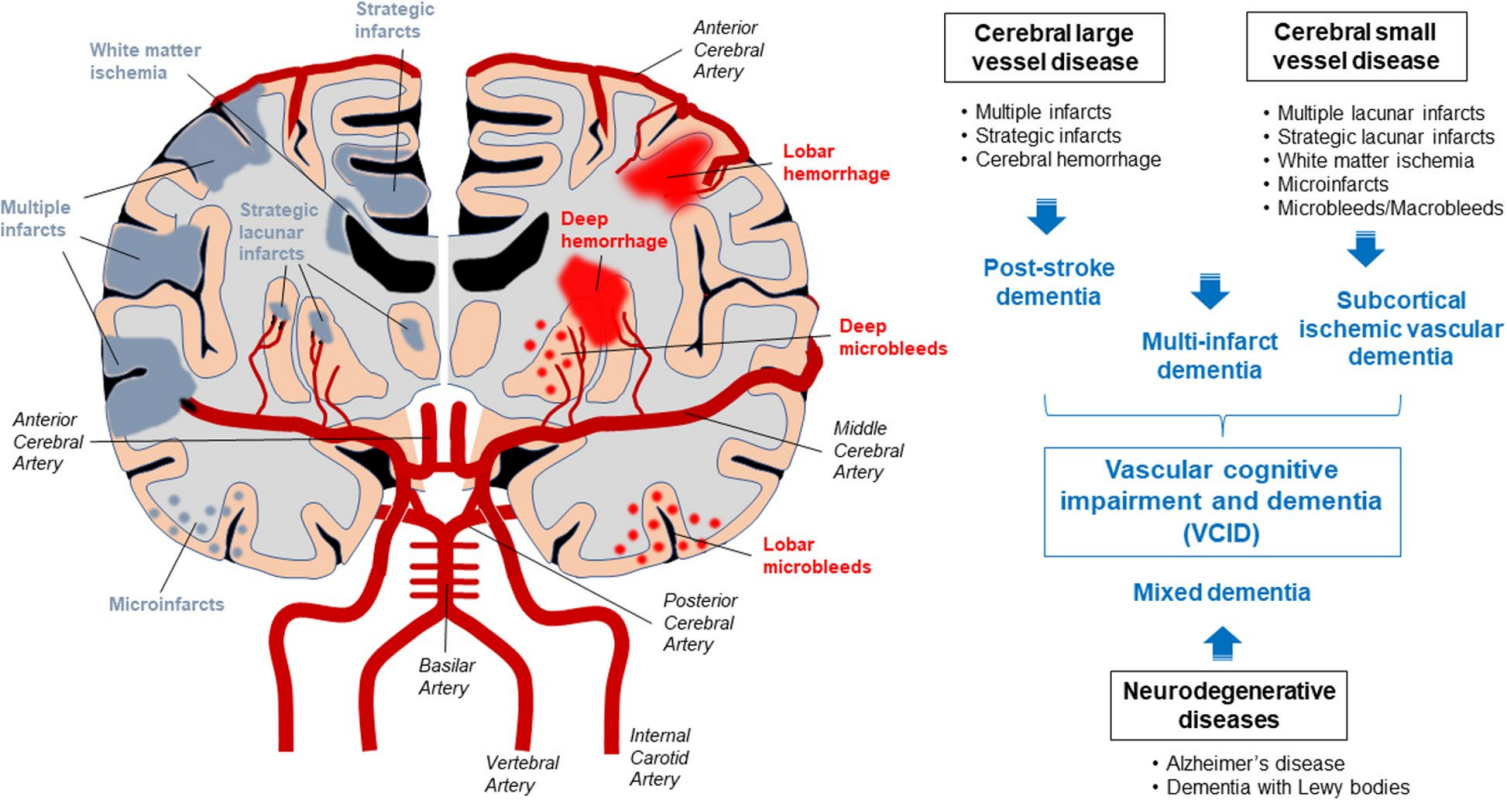
Cerebral vascular lesions and vascular cognitive impairment and dementia. Vascular cognitive impairment and dementia (VCID) is a major cause of age-related cognitive decline related to cerebrovascular damages in cerebral large and small vessels. The internal carotid arteries and the vertebral arteries mediate the arterial blood entry into the brain. The blood supply to the cerebrum is mediated by anterior cerebral arteries (ACA) and middle cerebral arteries (MCA) branched from internal carotid arteries. Posterior cerebral arteries (PCA) arising from vertebral arteries are responsible for the blood supply to the brainstem, cerebellum, and occipital cortex. Leptomeningeal arteries from the cerebral arteries form a network of vessels on the pial surface, which branch into the parenchyma. Based on vascular lesions, severe VCID is generally subtyped as post-stroke dementia, subcortical ischemic vascular dementia, multi-infarct dementia, and mixed dementia. Alzheimer’s disease often coexists with cerebrovascular lesions resulting in mixed dementia

The Vascular Impairment of Cognition Classification Consensus Study (VICCCS) identifies four major subtypes of vascular lesions that cause dementia: 1) Post-stroke dementia, 2) Subcortical ischemic vascular dementia, 3) Multi-infarct dementia, and 4) Mixed dementia [4] (Fig. 1). Post-stroke dementia is a major consequence after large vessel strokes. Approximately 10% of patients develop dementia after their first stroke [5]. Atherothrombotic brain infarcts [6] and hemorrhagic stroke [5, 7] are associated with the higher dementia risk. Subcortical ischemic vascular dementia is caused by stenosis and occlusion of small vessels that culminate into lacunar infarct and ischemic white matter lesions. Cortical-subcortical circuit disruption often leads to impairments in information processing, complex attention, and frontal-executive function [8]. Multi-infarct dementia refers to cognitive impairment due to multiple infarcts in various cortical arteries and arterioles. Cortical symptoms such as apraxia and aphasia are often diagnosed through cognitive function tests [2]. Mixed dementia is a type of dementia with concurrent vascular and neurodegenerative pathological changes [9]. AD pathology and cerebrovascular lesions frequently coexist in autopsy cases with dementia [10]. VCID preferentially impairs attention, executive function, and sparing memory [1]. However, cognitive impairments observed in both VCID and AD cases show similar age-associated comorbidities.

VCID is associated with heterogeneous pathological conditions in the cerebrovascular system, where cerebral small vessel disease (cSVD) is the most common pathology underlying VCID [11]. CSVD includes a heterogenous spectrum of pathological, clinical, and radiological cerebrovascular changes. In particular, vessel wall structure from leptomeningeal arteries and intraparenchymal arterioles (perforating arterioles and precapillary arterioles), capillaries, and venules often pathologically deteriorate [12, 13]. In this review, we summarize the current knowledge of cerebrovascular anatomy and cellular compositions, cSVD classification, neuroimaging characteristics, and risk factors. We also discuss probable etiology of cSVD as well as therapeutic strategies.

### Basic anatomy of cerebrovascular system

Two pairs of large arteries, the internal carotid arteries and the vertebral arteries, mediate arterial blood entry into the brain. Two vertebral arteries integrate into a basilar artery, which branches out to two posterior cerebral arteries (PCA), distributing blood supply to the brainstem, cerebellum, and occipital cortex. The circle of Willis is composed of pre-communicating segments of the right and left anterior cerebral arteries (ACA), connected via the anterior communicating artery. Pre-communicating segments of the right and left PCA are connected to their corresponding internal carotid arteries via the posterior communicating arteries. An ACA and a middle cerebral artery (MCA) arise from each internal carotid artery and are responsible for blood supply to the cerebrum (Fig. 1) [14]. Second-order branches from the cerebral arteries establish a network of vessels on the pia mater in the subarachnoid space [15]. On the pial surface, leptomeningeal arteries penetrate the pia mater and glia limitans from the subarachnoid space into the brain parenchyma, ramifying into arterioles and capillaries, which end as venules that flow back into the veins [16–18]. Since most arterioles do not form collateral networks, arteriole damage results in hypoperfusion and hypoxia of downstream vessels and their corresponding brain regions [19]. In addition, there are cortical watershed areas in border zones between ACA, MCA, and PCA territories [20]. While perforating arterioles from MCA and ACA ascend into deep brain territories including the basal ganglia and thalamic gray matter [21], border zones between penetrating arterioles and perforating arterioles in deep subcortical white matter regions near the lateral ventricle are known as the internal watershed area [20, 22]. Low perfusion pressure in watershed areas result in hemodynamic vulnerability [18].

**Fig. 2:**
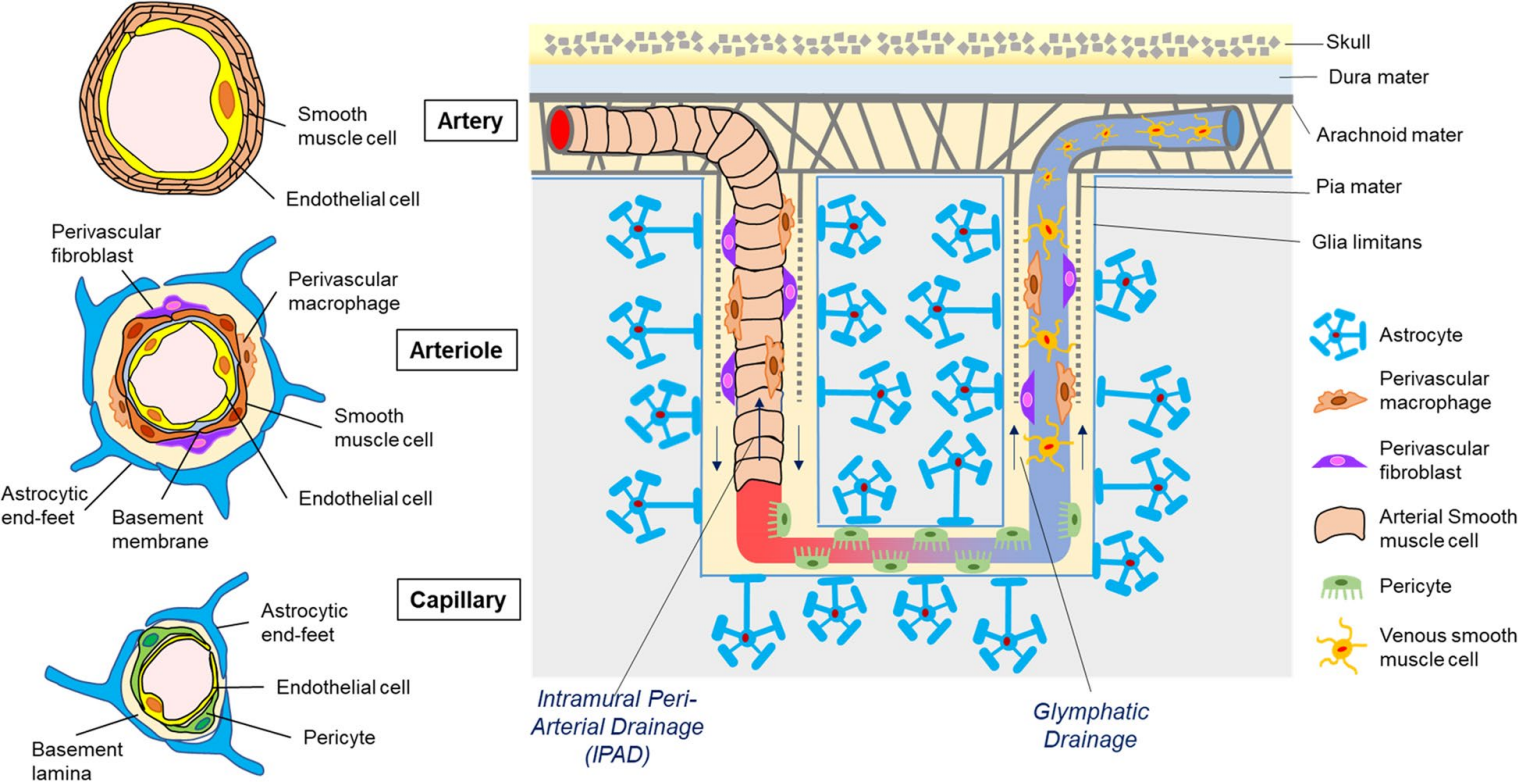
Structural and cellular compositions of the cerebral small vessels. Leptomeningeal arteries penetrate the pia mater and glia limitans from the subarachnoid space into the parenchyma, ramifying into arterioles and capillaries. In leptomeningeal arteries, endothelial cells make up a single luminal layer which are covered by multiple smooth muscle cell layers. In penetrating arterioles, endothelial cells and smooth muscle cells are single layers. In capillaries, endothelial cells form the blood–brain barrier (BBB) with pericytes and basement membrane, surrounded by astrocytic end-feet. The capillaries connect to venules flowing back into veins. In contrast to arterial smooth muscle cells, venous smooth muscle cells have flattened cell bodies and multiple processes, not fully sheathing the venules and veins. Perivascular fibroblasts and macrophages are mainly localized on arterioles and venules. Subarachnoid cerebrospinal fluid (CSF) distributes into the brain parenchyma through para-arterial spaces referred as glymphatic periarterial CSF influx. Interstitial fluid (ISF) as well as CSF diffuse into the perivenous space by bulk flow, and finally efflux into the CSF-dural sinus or cervical lymph nodes. In addition, the ISF and CSF in brain parenchyma can also enter the periarterial space from the capillary level and flow countercurrent to blood flow along arterial vessels, referred as intramural periarterial drainage (IPAD) pathway

Cerebral small vessels including penetrating arterioles, precapillary arterioles, postcapillary venules and venules are surrounded by perivascular or paravascular space filled with cerebrospinal fluid (CSF) and/or interstitial fluid (ISF) [23]. While pia mater coats the vascular walls and brain surface, they combine into a singular layer of pia that penetrates into the brain [24]. The vascular pial sheath internally borders the perivascular space. In contrast, the glia limitans (basement membrane of astrocyte end-feet) externally borders paravascular space [25]. While perivascular space is sometimes narrowly defined as the space in basement membranes between smooth muscle cell layers, the paravascular and perivascular spaces integrate in the capillaries due to the lack of smooth muscle layers and pial sheath [23, 26] (Fig. 2). ISF is predicted to enter the periarterial space through capillaries [27, 28], flow along the vessels to leptomeningeal arteries [29], and drain into cervical lymph nodes via the wall of the internal carotid artery, referred to as the intramural periarterial drainage (IPAD) pathway [29]. On the other hand, CSF influxes from the subarachnoid space into the brain parenchyma through paravascular spaces along arterial vessels, mixed with ISF and solutes in the brain parenchyma, drained into the perivenous tracts towards the subarachnoid space, dural venous sinuses, and dural lymphatic vessels for clearance. This pathway is known as glymphatic drainage (Fig. 2). However, further studies are needed to define the specific contributions of IPAD and glymphatic drainage to ISF/CSF clearance [30–33].

### Cellular components in cerebrovascular system

The endothelial cell layer (endothelium) is continuous along cerebral vessels. Although endothelial cell is the key cell type separating blood from brain tissue, other vascular cells make up cerebral vasculature: vascular mural cells (smooth muscle cells and pericytes), astrocytes, perivascular macrophages, and perivascular fibroblasts (Fig. 2) [34, 35]. Recent single cell or single nucleus RNA-sequencing studies show cell-specific gene expression heterogeneity at each cerebrovascular segment [36], albeit some discrepancies between methodologies and species [37, 38]. These vascular and perivascular cells interactively function in maintaining cerebrovascular homeostasis.

#### Endothelial cells at the blood–brain barrier (BBB)

Brain capillary formation through endothelial progenitor cell angiogenesis is mediated by vascular endothelial growth factor (VEGF) and Wnt signaling [39]. By interacting with pericytes and astrocytes, the brain capillary endothelial cells mature and create the tightly sealed monolayer with high barrier integrity, referred to as the BBB [40]. During BBB maturation, the brain capillary endothelial cells exhibit distinct properties from endothelial cells in other organs: specific tight junction protein expression, selective transporter expression, suppressed transcytosis, and leukocyte adhesion molecule downregulation [39, 41]. These unique endothelial properties allow the BBB to strictly control fluid and solute exchange between blood and the parenchyma. These endothelial cells are connected by specialized tight junction proteins such as occludins and claudins, forming a high resistance paracellular barrier [42–44]. Both occludin and claudin are tetraspan transmembrane proteins intracellularly linked to the actin cytoskeleton through zonula occludens-1 (ZO-1) [45, 46]. In addition to tight junctions, there are adherens junctions with vascular endothelial (VE)-cadherin, platelet endothelial cell adhesion molecule-1 (PECAM-1) and neural (N)-cadherin, and connexin 43 gap junctions between endothelial cells [47]. The basement membrane is composed of extracellular matrix proteins such as collagen type IV, heparan sulfate proteoglycans, and fibronectin that surround endothelial cells and pericytes. The basement membrane is also critically involved in BBB stability [48]. BBB tight junctions, adherens junctions, gap junctions, and the basement membrane prevent passive diffusion and passive paracellular transport from blood. Selective essential nutrients and metabolites exchange such as glucose, amino acids, fatty acids, organic anions, and nucleosides across the BBB are mediated via carrier-mediated transporters [48, 49]. ATP binding cassette (ABC) transporters are also expressed at the BBB as active efflux transporters to eliminate lipids and exogenous drug from the brain [50].

#### Vascular mural cells in the arteries and arterioles

In cerebral arteries and arterioles, endothelial cells and the internal elastic lamina layer structure (*tunica intima)* are surrounded by vascular smooth muscle cell layers (*tunica media*) and additional layers mainly composed of collagen fibers and fibroblasts (*tunica adventitia*) [51]. Leptomeningeal arteries contain several smooth muscle layers, which thin into a single layer in penetrating arterioles [36]. In precapillary arterioles, pericytes are the main vascular mural cell, sharing commonalities with smooth muscle cells [52]. Vascular smooth muscle cells are contractile cells responsible for controlling cerebral blood flow. Pericyte contribution to vascular contraction and blood flow regulation is controversial [52]; however, pericytes on first-order branches from penetrating arterioles appear to predominantly regulate capillary blood flow [53]. Since systemic blood pressure substantially influences brain circulation, vascular mural cells play a critical role in cerebral autoregulation through vascular tone modulation that maintains a relatively constant baseline cerebral blood flow [54]. Vascular tone is controlled by vascular mural cell membrane polarization through K^+^ channels and voltage-dependent Ca^2+^ channels. K^+^ channel depolarization (suppressed K^+^ efflux) and voltage-dependent Ca^2+^ channel opening (enhanced Ca^2+^ influx) induce vasoconstriction, while vascular mural cell membrane hyperpolarization causes vasodilation [55]. In addition, inositol 1, 4, 5-trisphosphate receptor (IP3R)–mediated Ca^2+^ release [56] and RhoA/Rho-kinase activation [57] contribute to vascular smooth muscle cell contraction.

#### Neurovascular coupling

Vascular mural cells relax in response to nitric oxide (NO), prostaglandins, epoxyeicosatrienoic acids (EET), adenosine triphosphate (ATP), and K^+^ released from neurons, astrocytes, and endothelia cells depending on neuronal activity, referred to as neurovascular coupling [58]. Neurovascular coupling increases blood supply to capillaries by 84% [59]. Neuronal glutamate activates phospholipase A2 (PLA2) through metabotropic glutamate receptors (mGluRs) and promotes astrocytic prostaglandin E2 (PGE_2_) and EET synthesis that result in vasodilation [60]. Glutamate also triggers intracellular Ca^2+^ influx through N-methyl-D-aspartate (NMDA) and α-amino-3-hydroxy-5-methyl-4-isoxazolepropionic acid (AMPA) receptors on the postsynaptic membrane. Cytosolic Ca^2+^ increases in astrocytic end-feet stimulate K^+^ efflux, inducing smooth muscle cell vasodilation [61]. However, excess astrocytic Ca^2+^ likely promotes vasoconstriction instead. NMDA and/or AMPA activation leads to neuronal NO synthase (nNOS) and cyclooxygenase 2 (COX-2) upregulation [62]. NO promotes smooth muscle cell vasodilation through cyclic guanosine monophosphate (cGMP)-dependent protein kinase (PKG) [63]. NO also inhibits vasoconstriction by impeding 20-hydroxyeicosatetraenoic acid (20-HETE) synthesis [59]. Inhibiting nNOS has been shown to reduce neurovascular response by 64% [64]. Neuronal COX facilities phospholipase I_2_ (PGI_2_) synthesis which in turn induces smooth muscle vasodilation through the cyclic adenosine monophosphate (cAMP)-protein kinase A (PKA) pathway [62, 65]. Controversially, Neuropeptide Y (NPY) released after inhibitory neuron activation likely induces vasoconstriction [66]. In the hypothalamic supraoptic nucleus (SON), vasopressin (VP) neuronal activation also causes responsive vasoconstriction upon acute salt loading challenge [67].

Angiotensin II has been shown to disrupt neurovascular coupling by increasing Ca^2+^ through angiotensin II type 1 (AT1) receptor in the nearby astrocytic end-feet [68]. In addition, endothelial cells are involved in NO-mediated vasodilation through endothelial NO synthase (eNOS) regulation via NMDA receptor signaling [69] or upon mechanical shear stress [70]. Hypoxia, thrombin, and inflammatory cytokines promote the production of endothelin (ET), a strong vasoconstrictor, in endothelial cells [71]. Angiotensin II also promotes peroxynitrite (ONOO^−^) generation through the AT1-NADPH oxidase (NOX) pathway in endothelial cells, resulting in neurovascular coupling impairment (Table 1) [72].

**Table 1:** Cell type-specific regulation of vasodilation and vasoconstriction

Vascular cell types	Vasodilation	Vasoconstriction
Vascular mural cells	• Hyperpolarization: K^+^ channels ↑, Ca^2+^ channels ↓[55] • Activation of NO-cGMP-PKG pathway [63] • Activation of cAMP-PKA pathway [65]	• Depolarization: K^+^ channels ↓, Ca^2+^ channels ↑[55] • Cytosolic Ca^2+^ increase mediated by IP3 receptor [56] • Activation of RhoA/Rho-kinase pathway [57]
Neurons	• NMDA and AMPA glutamate receptor-dependent: ◦ NO release through nNOS [62] ◦ PGI_2_ synthesis [62]	• NPY release from activated inhibitory neurons [66] • VP release from VP neurons in the SON [67]
Astrocytes	• mGluR-dependent PGE_2_ and EET synthesis [60] • Astrocytic end feet K^+^ release in response to increased cytosolic Ca^2+^ [61]	• Excess increase of cytosolic Ca^2+^ induced by Angiotensin II through AT1 receptor [68]
Endothelial cells	• NO release through eNOS induced by: ◦ Glutamate signaling via NMDAR [69] ◦ Shear stress [70]	• ET release induced by hypoxia, thrombin, and inflammatory cytokines [71] • ONOO^−^ production by Angiotensin II through AT1-NOX pathway [72]

#### Pericytes on the capillaries

Pericytes are localized at the abluminal side of the capillary endothelial cells and form direct synaptic-like peg-socket focal contacts with endothelium through N-cadherin and connexins [73]. Pericytes cover capillary endothelial cells with a 1:3 pericyte-to-endothelium ratio [74]. Pericytes contribute to various aspects of cerebrovascular functions including angiogenesis, BBB integrity [47, 75], and immune cell filtration [76] through the crosstalk with endothelial cells, astrocytes, neurons, and microglia [77]. Pericytes secrete VEGF to promote angiogenic sprouting and stabilization of endothelial cells [78]. Platelet-derived growth factor-BB (PDGF-BB) secreted from endothelial cells also critically mediates pericyte angiogenesis through PDGF receptor-β (PDGFRβ) [79–81]. Pericyte–endothelial signals, including PDGF-BB–PDGFRβ, VEGF–VEGF receptor-2 (VEGFR2), transforming growth factor-β (TGF-β)–TGF-β receptor 2 (TGFβR2), Angiopoietin (Ang)-Tie2, Notch, and major facilitator superfamily domain-containing 2a (MFSD2A), play an important roles in BBB development, maintenance of integrity, and transport [82]. Pericytes also modulate brain immune responses [76]. In vivo studies in animals have demonstrated that loss of pericytes lead to upregulation of leukocyte adhesion molecules (LAMs) on endothelial cells, exacerbating parenchymal immune cell infiltration [79, 80].

#### Astrocytes in the glial limitans and BBB

The glia limitans is composed of astrocytic end-feet and the basement membrane. The glia limitans constitute a continuous layer covering a large area of cerebral small vessels as external limitans of perivascular/paravascular space and mediates the ISF and CSF transport between the brain parenchyma and drainage pathways [83]. Leptomeningeal arteries and penetrating arterioles are covered by pia mater and glia limitans superficialis. In capillaries, glia limitans perivascularis surround endothelial cells and pericytes [84]. Astrocytes secrete vasculotrophic factors such as astrocyte-derived angiopoietin-1 (ANG-1), sonic hedgehog (SHH), glial-derived neurotrophic factor (GDNF), retinoic acid (RA), insulin-like growth factor-1 (IGF-1) and apolipoprotein E (APOE) involved in maintaining BBB integrity [85]. Supporting this, experiments co-culturing astrocytes and endothelial cells show upregulated expression of tight junction proteins and strengthened barrier integrity [86]. Astrocytic end-feet also express α-dystroglycan to anchor basement membrane and maintain BBB function [87]. Furthermore, water channel aquaporin 4 (AQP4) expressed on astrocytic end-feet plays an essential role in regulating cerebral water homeostasis and glymphatic drainage [88, 89].

#### Perivascular cells on cerebral vessels

Perivascular macrophages are yolk sac-derived immune cells detected on brain arterioles and venules [90]. Perivascular macrophages localize in perivascular or paravascular space under the glial limitans, mediating brain immune responses through phagocytosis and antigen presentation [34]. Perivascular macrophages are also involved CSF flow and glymphatic drainage regulation. A study in mice has shown that depletion of perivascular macrophages causes excess accumulation of extracellular matrix proteins in perivascular/paravascular space, thereby disturbing CSF perfusion [91].

Perivascular fibroblasts are identified as cells with flattened somata and sheet-like ruffled processes on penetrating arterioles, precapillary arterioles, and ascending venules in the brain [92]. Although physiological roles of perivascular fibroblasts remain unclear, perivascular fibroblasts appear to serve as tissue-resident mesenchymal cells [93]. It is predicted that these cells maintain the vascular basement membrane, glymphatic drainage system, and mechanosensation for neurovascular coupling [35]. Upon tissue damage, the perivascular cells are the likely major source for scar formation and fibrosis by producing extracellular matrix proteins and mediating inflammation [93].

### Classifications of cSVD

Clinically cSVD shows progressive symptoms in cognitive impairment, depression, urinary disturbance, gait difficulty, dysphagia, and dysarthria [13]. While cSVD has relatively homogenous clinical features, six types of cSVD have been proposed based on etiopathogenic features: 1) Arteriolosclerosis, 2) Sporadic and hereditary cerebral amyloid angiopathy (CAA), 3) Inherited or genetic cSVD distinct from CAA, 4) Inflammatory and immunologically mediated cSVD, 5) Venous collagenosis, and 6) Other cSVD [13].

#### Arteriolosclerosis

Arteriolosclerosis is the most common form of cSVD, neuropathologically defined by hyaline thickening of vessel walls (< 150 μm in diameter) without association with lipid-containing cells, intramural inflammation, and amyloid or fibrinoid necrosis [94]. Smooth muscle cell loss from the *tunica media* and deposits of fibro-hyaline material and collagens in vessel walls are also detected in arteriolosclerosis lesions [13]. The Vascular Cognitive Impairment Neuropathology Guidelines (VCING) system has been widely used to evaluate the severity of arteriolosclerosis in a semiquantitative manner; 0 = Normal, 1 = Mid thickening of the vessel media with mid fibrosis, 2 = Partial loss of smooth muscle cells in the media with moderate hyaline fibrosis, and 3 = Complete loss of smooth muscle cells in the media with severe hyaline fibrosis and lumen stenosis [94]. Arteriolosclerosis is also exacerbated by diabetes and hypertension during aging [95]. Fibrinoid necrosis commonly accompanies arteriolosclerosis in hypertensive arteriopathy [96–98]. Other pathological microangiopathies include microatheroma (distal manifestations of atherosclerosis) and microaneurysms (elongated and dilated vessels) [13]. Of note, cerebral arteriolosclerosis has been reported as a predominant factor contributing to global cognitive impairments, episodic memory, working memory, perceptual speed, autonomic dysfunction, and motor symptoms [99].

#### Cerebral amyloid angiopathy

CAA is characterized by the progressive accumulation of amyloid-β (Aβ) in leptomeningeal arteries, penetrating arterioles, and capillaries. Aβ deposits begin at the basement membrane between smooth muscle cell layers and develop into circumferential transmural deposits [100]. Vessel integrity loss caused by Aβ deposits can lead to spontaneous lobar intracerebral hemorrhage [101]. Aβ also disrupts the vascular extracellular matrix layers, causing luminal obstruction, leading to parenchymal ischemia [102]. Population-based postmortem studies demonstrated that CAA is detected in 20–40% of elderly people without dementia and 50–60% of those with dementia [103–106]. CAA is associated with cSVD neuroimaging markers on magnetic resonance imaging (MRI), including microbleeds [107], white mater hyperintensities (WMHs) [108], and microinfarcts [109]. Of these, lobar cerebral microbleeds is strongly predictive of CAA [110]. CAA-related microbleeds are frequently identified at the gray-white matter junction of the parietal and occipital lobes [102]. CAA categorization heavily relies on intracerebral hemorrhage status to define individual cases as “definite CAA”, “probable CAA with supporting pathological evidence”, “probable CAA”, or “possible CAA” under the modified Boston criteria [111, 112] and Boston criteria version 2.0 [113]. Another non-hemorrhagic neuroimaging marker, perivascular spaces at the centrum semiovale, reflects perivascular interstitial fluid drainage impairments [114]. Subcortical WMHs [115] and posterior predominant WMHs are also observed in CAA [116]. CAA-related WMHs are likely caused by hypoperfusion associated with Aβ deposits in cortical small vessels, BBB disruption, and following increases in vascular permeability [101, 117]. WMH severity is associated with a higher risk of recurrent lobar hemorrhage, larger hematoma volume, and hematoma expansion [118, 119]. Cerebral microinfarcts are acute or subacute ischemic infarctions observed in patients with advanced CAA [109]. They appear as round or oval white colored areas that indicate high intensity regions in the subcortex and cortex on diffusion-weighted MRI [109]. CAA also causes convexity subarachnoid hemorrhage and transient focal neurological episodes (TFNEs) [120]. TFNEs are short, stereotyped episodes of somatosensory or motor disturbance, dysphasia, and visual loss, often accompanied with cortical spreading, depression, or depolarization due to the superficial hemorrhage [121].

#### Inherited or genetic cSVD

The Cerebral Autosomal Dominant Arteriopathy with Subcortical Infarcts and Leukoencephalopathy (CADASIL), caused by a mutation in *NOTCH3,* has been known as one type of hereditary cSVD [122]. Clinical features of the mutation carriers include migraines with aura, recurrent ischemic strokes, transient ischemic attacks with cognitive impairment, and subcortical dementia [123]. Brain MRIs show hyperintense periventricular lesions and centrum semioval on T2-weighted or fluid attenuation inversion recovery (FLAIR) images [123]. This progresses to confluent leukoaraiosis with anterior temporal lobe involvement [124]. Pathological analyses show granular osmiophilic material (GOM) in the *tunica media* and vessel wall thickening [125]. Another form of hereditary cSVD is Cerebral Autosomal Recessive Arteriopathy with Subcortical Infarcts and Leukoencephalopathy (CARASIL). CARASIL is caused by mutations in the *HTRA1* gene that encodes HtrA serine peptidase/protease 1 (HTRA1) [126]. Clinical features include early-onset lacunar stroke, cognitive impairment, alopecia, and lumbar spondylosis [127]. Lacunar stroke in the basal ganglia or brainstem is the most common manifestation of CARASIL, observed in approximately 50% of cases. Extensive vascular smooth muscle cells degeneration, vessel wall thickening, and lumen narrowing are histologically observed in CARASIL [125, 128]. Missense or null variants in *COL4A1* and *COL4A2* result in autosomal dominant cSVD [129]. These mutations are accompanied with cerebral microbleeds in the basal ganglia, centrum semiovale, and pons, and/or small deep lacunar infarcts and dilated perivascular spaces in the basal ganglia [130]. *COL4A1* and *COL4A2* mutations also induce other clinical manifestations in the brain (porencephaly, and intracerebral aneurysms), eyes (cataracts, retinal vascular tortuosity, and retinal hemorrhage), and kidneys (proteinuria, renal insufficiency, renal cysts, and tortuosities of retinal arteries) [131, 132]. Hereditary diffuse leukoencephalopathy with spheroids (HDLS) caused by *CSF1R* mutations is an early-onset dementia with brain atrophy and white matter changes [133]. HDLS is characterized by WMHs with frontal or frontoparietal predilection and asymmetric distribution, brain atrophy, and corpus callosal involvement [134]. Clinically, HDLS show symptoms related to frontal lobe syndrome such as loss of judgment, lack of social inhibition, lack of insight, and personality changes [135]. Mitochondrial encephalomyopathy, lactic acidosis, and stroke-like episodes (MELAS) syndrome is a maternally inherited mitochondrial disorder with the m.3243A > G variant that results in multi-organ dysfunction [136]. The clinical MELAS manifestations are varied including stroke-like episodes, dementia, epilepsy, lactic acidosis, myopathy, hearing impairment, diabetes, headache, and short stature. Stroke-like episodes are frequently observed in occipito-temporal regions, presenting as vasogenic edema in the acute phase [137]. Fabry’s disease is an X-linked, recessive lysosomal storage disease affecting glycosphingolipid metabolism, caused by a mutation in *GLA* which encodes alpha-galactosidase A (α-Gal-A). The clinical symptoms include peripheral polyneuropathy, autonomic dysfunction, and posterior circulation strokes [138].

#### Inflammatory and immunologically mediated cSVD

This group of cSVD is characterized by excess immune cell infiltration into the vessel walls (vasculitis) due to systemic and vascular inflammation during infection, autoimmune diseases, and rare immunological diseases [13]. A community-based population study showed that high neutrophil count is associated with increased risk for enlarged perivascular spaces in the basal ganglia and lacune [139]. Plasma C-reactive protein (CRP) or interleukin 6 (IL-6) levels were also positively correlated with the presence of WMHs [140, 141].

#### Venous collagenosis

Venous collagenosis is noninflammatory collagenous thickening of venous walls mainly composed of collagen I and III in white matter regions along lateral ventricles. A histological study found venous collagenosis in 65% of cases in an over 60-year-old cohort [142]. Venous collagenosis is associated with leukoaraiosis severity or periventricular white matter ischemia [142]. Interestingly, increased venous collagen, but not arterial collagen, is reported as a significant predictor of higher WMH burden [143]. Since venous collagenosis also causes luminal stenosis or occlusion, venous lesions are predicted to associate with cerebral hypoperfusion, glymphatic drainage disruption, and BBB damage [144].

#### Other cSVD

Although radiotherapy is effective in treating cancers, cranial radiation sometimes causes irreversible cerebrovascular damage in delayed phases. It includes arteritis, intracranial aneurysm, cavernous malformation, mineralizing microangiopathy [145]. Endothelial cells and neurons are vulnerable to radiation, and cSVD is also a complication after radiation therapy. CSVD including microbleeds, microinfarcts, or white matter lesions are often observed in long term follow-up after cranial irradiation [146].

### Neuroimaging hallmarks of cSVD

The STandards for Reporting and Imaging of Small Vessel Disease (STRIVE) guideline defines WMHs, recent small subcortical infarcts, lacunes, cerebral microbleeds, enlarged perivascular spaces, and brain atrophy which are common features of cSVD detected through neuroimaging [147]. These MRI hallmarks can become apparent long before symptom onset as clinically silent manifestations [13]. Such lesion accumulations subsequently lead to an increased risk of stroke [1, 21, 148], depression, and mobility disorders as well as VCID [149]. Here, we summarize MRI features of cSVD.

#### White matter hyperintensities (WMHs)

WMHs are frequently detected in VCID patients. A population-based study reported that WMHs are 95% pervasive in the elderly population over 60 years old [150]. WMH progression is associated with executive function, attention, and immediate/delayed memory [151]. WMHs are lesions detected as hyperintense areas on T2 or FLAIR [152]. Deep WMHs are often smaller and asymmetrically distributed in juxtacortical white matter. This distribution is suggestive of local perfusion impairments due to hypertensive arteriopathy and CAA [153]. Periventricular WMHs are located symmetrically around ventricles, suggesting occlusive periventricular venous collagenosis-related diffuse perfusion disturbances [142, 152, 154]. Pathological examination of WMHs shows varied degrees of demyelination, diffuse axonal injury, gliosis, and oligodendrocyte loss [94, 155]. In deep WMHs, hypoxia-inducible factor (HIF) levels are upregulated in cerebral capillary endothelial cells, supporting ischemic associations [156]. Furthermore, BBB disruption characterized by activated astrocytes and fibrinogen positivity is also associated with both deep and periventricular WMHs [157].

#### Recent small subcortical infarcts

Recent small subcortical infarcts, commonly called lacunar infarction, account for about 25% of all ischemic strokes [158]. Lacunar infarcts are defined by neuroimaging evidence of recent infarction around a single perforating arteriole with a diameter of less than 20 mm in axial section. “Recent” refers to symptoms or imaging features formed during the hyperacute phase and the first few weeks before diagnostic imaging [147].

#### Lacunes of presumed vascular origin

Lacune is used to describe round or ovoid, subcortical, fluid-filled cavities with a diameter of 3–15 mm, formed during healing from lacunar infarcts or small hemorrhages. Lacune prevalence range from 8–28% (mean age: 50–75 years) [159]. Increased lacune counts are associated with a higher risk of cognitive impairment and stroke [159, 160].

#### Enlarged perivascular space

In axial MRI imaging, enlarged perivascular spaces are observed as hyperintense round lesions surrounding perforating arteries and arterioles in the basal ganglia and linear in the centrum semiovale on T2-weighted images. Mechanisms underlying enlarged perivascular spaces is not well understood. However, it is hypothesized to represent perivascular fluid stagnation due to lymphatic drainage blockage [161]. Larger numbers of enlarged perivascular spaces are associated with worsened cognitive function or dementia [162].

#### Microbleeds

The prevalence of cerebral microbleeds is between 11.1–15.3%, increasing in an age-dependent manner [163, 164]. Cerebral microbleeds are MRI-visible small hypointense oval or round lesions with a diameter of 2–10 mm detected through T2* weighted gradient imaging [165] or magnetic susceptibility weighted images [165]. Cerebral microbleeds are perivascular hemosiderin deposits that reflect previous subtle hemorrhages from small vessels involved in arteriolosclerosis or CAA [147, 166]. Lobar cerebral microbleeds in cortico-subcortical areas are associated with cognitive impairment [167–169] and lobar intracranial hemorrhage [170]. Deep/infratentorial cerebral microbleeds are detected as hypertensive vasculopathy in deep gray or white matter of the cerebral hemispheres, brainstem, and cerebellum [171].

#### Brain atrophy

Brain atrophy manifests as general or focal, and symmetrical or asymmetrical decrease of gray or white matter volumes. Brain atrophy frequently occurs with increased ventricular volumes, enlarged superficial sulci, and WMHs. Some studies show that increased WMHs further aggravate brain atrophy [172, 173].

### Risk factors for cSVD

Collective evidence suggests that age, hypertension, diabetes, hyperlipidemia, smoking, and obstructive sleep apnea substantially impact cSVD pathogenesis [174–176]. Among them, aging and hypertension are the predominant factors associated with cSVD risk [177]. Epidemiological studies show that cSVD prevalence is higher in cases with longstanding hypertension in middle age [178, 179]. In addition, recent reports indicate that a subset of COVID-19 patients have neuroimaging features of cSVD [180, 181]. In this section, we summarize the pathogenic conditions that influence cSVD risk and genetic risk factors.

#### Aging

Aging is involved in various pathogenic conditions, including hypertension, hyperlipidemia, diabetes, cardiovascular diseases, and dementia [182]. A meta-analysis study found that BBB permeability increases with age in both healthy and demented individuals [183]. A neuropathological study also showed that age exacerbates cSVD score in AD brains [184]. While WMH burden increases with age, age-dependent effects are accelerated in the presence of hypertension, abnormal body mass index (BMI), and diabetes mellitus after 50 years of age [185]. Overall prevalence of cerebral microbleeds was high and increased with age from 17.8% in persons aged 60–69 years to 38.3% in those over 80 years [171]. Thus, aging contributes to cSVD risk as a predominant factor. Although various pathogenic mechanisms such as oxidative stress, mitochondrial dysfunction, and chronic inflammation contribute to age-related vascular dysfunction [182], vascular senescence is also a critical cause compromising cerebrovascular function [186].

#### Hypertension

Hypertension is a leading risk factor for cSVD [13, 147] and VCID [187], which critically contributes to disease pathogenesis. WMH severity has a positive linear correlation with blood pressure [188]. A population-based study found that premorbid systolic blood pressure preceding 20 years before neuroimaging is more predominantly associated with cSVD burden than current systolic blood pressure [189]. Hypertension is also the most consistent predictor of cerebral microhemorrhage in healthy adult individuals and stroke patients [165]. While blood pressure variability has been shown to correlate with cardiovascular disease risk [190], it is also involved in cSVD development. Larger variation in systolic blood pressure in midlife is related to WMH development and ventricular atrophy later in life [191]. Hypertension damages cerebral vessels through multiple mechanisms: suppression of NO production, induction of reactive oxygen species (ROS), and extracellular matrix remodeling [192, 193].

#### Diabetes mellitus

Diabetes mellitus is a well-established risk factor for developing ischemic, hemorrhagic stroke, dementia, and cardiovascular diseases [194, 195]. Type 2 diabetes mellitus is associated with a higher risk of lacunar occurrence, although its impact on WMHs is controversial [196–198]. Since increased cSVD burden is detected in type 2 diabetes patients with retinopathy compared with those without retinopathy, small vessels are likely damaged in multiple organs during diabetes [199]. Hyperglycemia, insulin resistance, and altered fatty acid metabolism accompanied with diabetes mellitus have been known to induce oxidate stress and activation of the PKC pathway and receptors for advanced glycation endproducts (RAGE). These factors lead to suppression of NO production, inflammation, and thrombosis activation, resulting in substantial damages in endothelial and vascular mural cells [200].

#### Smoking

Smoking is known to cause deleterious effects on the vascular system, resulting in coronary heart disease, hypertension, arteriosclerosis, and stroke [201]. A meta-analysis showed that stroke morbidity and mortality are significantly higher in ever smoker groups with OR 1.45 and OR 1.44, and in current smoker groups with OR 1.90 and OR 1.70, compared to non-smoker groups [202]. A dose-dependent negative association between cigarette smoking and cortical thickness has also been reported [203]. Smoking is a strong factor associated with increased cSVD burden [204]. While smoking exacerbates WMHs [205] and white matter microstructural integrity [206], the effects on microbleeds, lacunes, and perivascular space enlargement are controversial [159, 207]. Toxic effects of cigarette smoking on endothelial cells are mainly induced by oxidative stress initiated by ROS, reactive nitrogen species, and other oxidant constituents [208]. Smoking also activates immune cells and induces vascular inflammation including leukocyte infiltration, matrix metalloproteinase (MMP) upregulation, and platelet/coagulation activation [208].

#### Obstructive sleep apnea

Obstructive sleep apnea are recurrent breathing interruptions during sleep [209] and is a strong risk factor for vascular diseases including hypertension, atherosclerosis, cardiovascular disease, and stroke [210–212]. As acute sleep deprivation decreases regional cerebral blood flow in healthy individuals [213], sleep fragmentation is related to the severity of cSVD neuroimaging markers [214]. Consistently, several studies show a significant association of obstructive sleep apnea with WMHs, but not microhemorrhage [215]. Obstructive sleep apnea causes ischemia/reperfusion injury [216, 217], where increased ROS and pro-inflammatory molecules cause brain damage [218]. Furthermore, accumulating evidence indicates that sleep disturbances compromise glymphatic drainage [219]. The severity of obstructive sleep apnea is associated with enlarged perivascular space in the basal ganglia and centrum semiovale [215].

#### Hyperlipidemia

Healthy individuals with dyslipidemia (total triglyceride > 150 mg/dL and/or high-density lipoprotein [HDL] < 40 mg/dL) are associated with subcortical WMHs [220]. Another study also demonstrated that total triglyceride levels, but not low-density lipoprotein (LDL) or HDL, were associated with larger WMH volume and lacune [221]. Controversially, there is a report showing that ischemic stroke patients with a history of hyperlipidemia (total cholesterol > 220 mg/dL or total triglyceride > 150 mg/dL, and prescription of statin) have less severe WMHs [222]. Higher total cholesterol (200–225 mg/dL) is significantly associated with a lower cSVD risk such as lacunar infarctions and WMHs as detected by MRI in a middle-aged population who visited a hospital for a brain checkup [223]. Although a meta-analysis showed the positive correlation between hyperlipidemia and cSVD risk [174], it remains controversial. Thus, hypertriglyceridemia but not hypercholesterolemia may be associated with increased cSVD risk as hypertriglyceridemia compromises endothelial function by causing oxidative stress [224]. Higher blood LDL is also associated with exacerbated AD neuropathology [225].

#### COVID-19

COVID-19 due to SARS-CoV-2 infection has substantially impacted the population health worldwide since December in 2019. In severe COVID-19 cases, there is respiratory failure and systemic inflammation. COVID-19 patients frequently show neurological symptoms including encephalopathy, encephalitis, and ischemic stroke [226]. Of note, a prospective study enrolling 60 recovered COVID-19 patients demonstrated that neurological symptoms are present in 55% of cases. Neuroimaging found micro-structural and functional brain integrity disruption during COVID-19 recovery [227]. Several case reports also indicate an association between COVID-19 and cSVD [228, 229]. While ischemic stroke occurs during the acute phase of COVID-19, cSVD phenotypes such as microinfarctions and vessel wall contrast enhancement are detected in the later phase [230]. In addition, COVID-19 has been reported to cause cerebrovascular endothelial loss, increasing the number of thin collagen IV-positive vessels lacking endothelial cells [231]. When SARS-CoV-2 infects endothelial cells, a viral protease (M^pro^) appears to reduce nuclear factor (NF)-κB essential modulator (NEMO) and suppress the receptor-interacting protein kinase 3 (RIPK3) pathway, leading to endothelial apoptosis and BBB breakdown [231]. Although further studies in larger cohorts are required, COVID-19 is likely associated with a higher risk for cSVD compared to other infective diseases.

#### Socioeconomic disparities

Several studies demonstrate that socioeconomic disparities are associated with stroke incidence, outcomes, and recurrence [232–234]. Other factors associated with lower socioeconomic status such as substance dependence, mental illness, and infectious diseases may contribute to pathogenesis. Consistently, socially marginalized individuals have shown higher prevalence (32%) for cSVD and often at a younger age (median 44.7 years old) [235]. Higher prevalence of WMHs is also associated with alcohol consumption [236], nonprescribed drug usage [237, 238], and nutritional deficiencies [239].

#### Genetic factors

Recent genome-wide association studies (GWAS) identified 31 loci associated with cSVD-related imaging traits including WMHs, mean diffusivity, and fractional anisotropy in 42,310 individuals. CSVD risk loci include gene coding proteins related to AD (*APOE* and *MAPT*), immune system (*HLA-B* and *HLA-S*), and extracellular matrix (*COL4A2* and *VCAN*) [240]. Although *APOE*-ε2 is protective against AD, both *APOE*-ε2 and ε4 have been known to increase CAA and CAA-related hemorrhagic risk [241, 242]. *APOE*-ε2/ε4 carriers are prone to developing CAA at an early age [243]. *APOE*-ε2 and ε4 are associated with CAA in arteries/arterioles and capillaries, respectively [244]. In addition to CAA, a meta-analysis implies that *APOE*-ε2 and ε4 are associated with increased WMH burden. While *APOE*-ε4 is correlated with lobar microbleeds, *APOE*-ε2 increases the risk of brain infarct [245].

### Probable etiology of cSVD

**Fig. 3:**
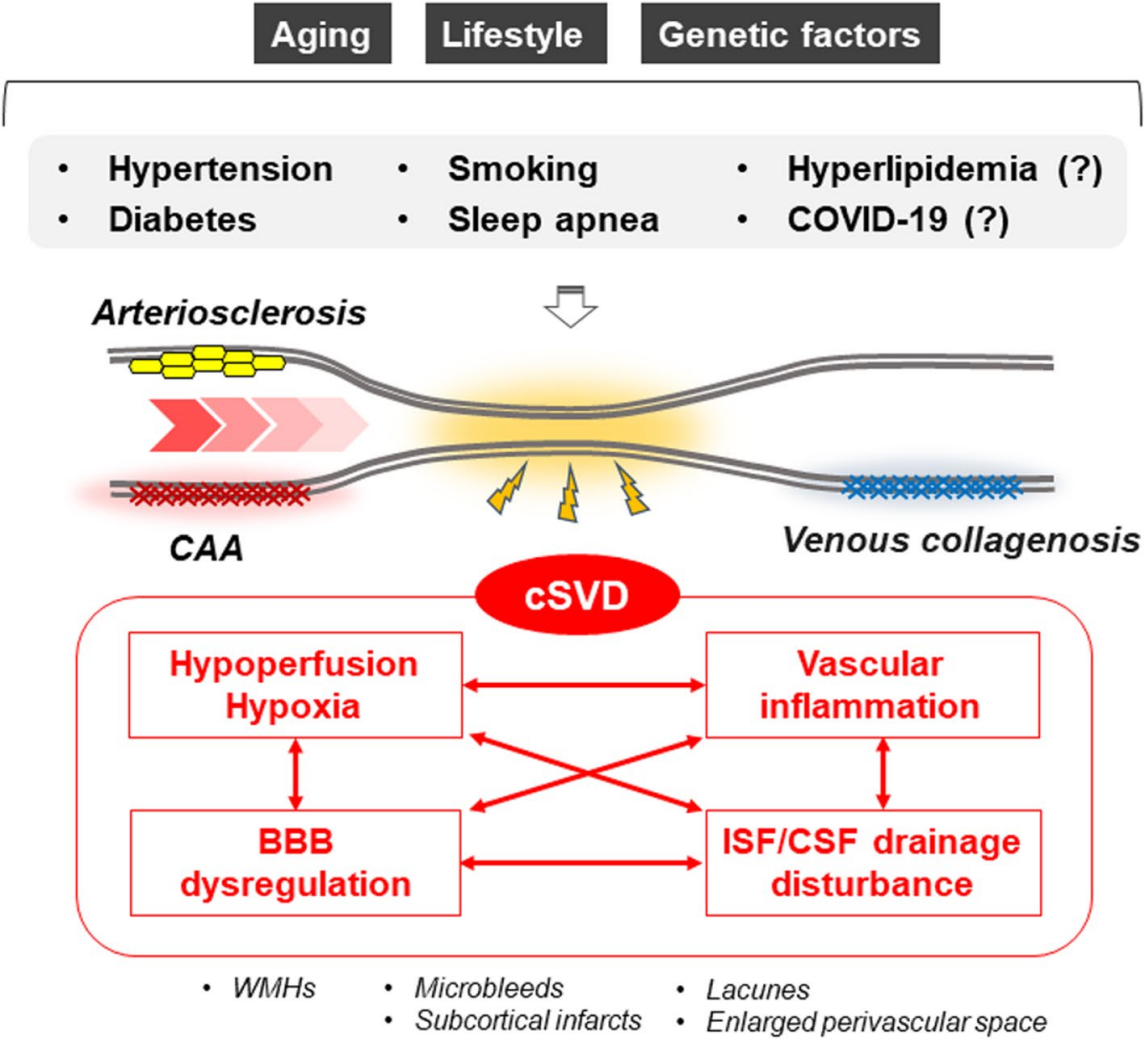
Risk factors and pathogenic mechanisms of cerebral small vessel disease. The cerebral small vessel disease (cSVD) can be classified into six groups including arteriolosclerosis, sporadic and hereditary cerebral amyloid angiopathy (CAA), inherited or genetic cSVD distinct from CAA, inflammatory and immunologically mediated cSVD, venous collagenosis, and others. Neuroimaging hallmarks of cSVD include white matter hyperintensities (WMHs), microbleeds, subcortical infarcts, lacunes, and enlarged perivascular space. While various molecular mechanisms are involved in cSVD pathogenesis, hypoperfusion/hypoxia, blood–brain barrier (BBB) dysregulation, interstitial fluid (ISF)/cerebrospinal fluid (CSF) drainage disturbances, and vascular inflammation are likely the major etiological pathways. Hypertension, smoking, diabetes, and sleep apnea are strongly associated with the risk of cSVD, where aging, lifestyle, and genetic factors also contribute to the pathogenic pathways as modifiers

The etiological mechanisms of cSVD can be summarized in the following four pathways: 1) hypoperfusion/hypoxia, 2) BBB dysregulation, 3) ISF/CSF drainage disturbances, and 4) vascular inflammation (Fig. 3) [246]. Each of them is predicted to contribute independently and interactively to cSVD pathogenesis.

#### Hypoperfusion/hypoxia

The brain needs a constant supply of oxygen and nutrition from blood to maintain the cellular and functional homeostasis. Arteriosclerosis, CAA, venous collagenosis, and other pathological changes detected in cSVD might cause not only luminal narrowing but also dysregulation of cerebral autoregulation, resulting in the reduction of cerebral blood supply. Capillary endothelial cells are vulnerable to elevated shear stress and hypoperfusion [247]. Chronic cerebral hypoperfusion and subsequent intermittent hypoxia provoke oxidative stress, mitochondrial dysfunction, inflammation, and proteinopathy, leading to neurodegeneration [248]. Particularly, white matter is susceptible to hypoperfusion/hypoxia due to poor collateral flow [249]. Cross-sectional studies demonstrated that lower cerebral blood flow is associated with higher WMH burden [250]. It is possible that brain atrophy due to white matter damage cause cerebral blood flow reduction [251]. However, a population-based study showed that lower cerebral perfusion at the baseline is associated with accelerated cognitive decline during follow-up. Animal studies also demonstrated that chronic hypoperfusion leads to white matter injury, lacunar infarcts, hemorrhages, and cognitive impairment, further exacerbated by *APOE*-ε4 [252]. Consistently, carotid revascularization has proved to improve cognitive function in patients with severe carotid stenosis by ameliorating cerebral hypoperfusion [253]. While the cascade to cSVD might vary depending on subtype, cerebrovascular hypoperfusion is predicted to predominantly trigger the etiological pathway [254, 255].

#### BBB dysregulation

Altered paracellular and transcellular transport, decreased tight junction proteins, basement membrane abnormality, and pericyte dysfunction characterize BBB dysregulation, which lead to aggravated plasma protein leakage and leukocyte infiltration into the brain parenchyma resulting in glial activation, demyelination, and neurodegeneration [50]. Neuroimaging studies have demonstrated greater BBB leakage in regions with WMH than normal-appearing white matter, which positively correlates with WMH severity, age, and hypertension [256, 257]. BBB leakage in WMH likely proceeds cognitive decline. As such, BBB dysregulation is causatively involved in cSVD symptoms. Notably, chronic cerebral hypoperfusion or hypoxia is a major factor causing BBB damage. For example, HIF-1 upregulates VEGF in pericytes and astrocytes during hypoxic conditions [258–260]. Excessive VEGF exacerbates BBB leakage through altering the distribution of tight junction proteins [261], despite VEGF having beneficial effects including collateral vessel formation, reparative angiogenesis, and neuroprotection after ischemic stroke [262]. Cerebral hypoperfusion also reduces capillary pericyte coverage, disrupting BBB integrity [78]. CSVD risk factors such as hypertension, diabetes, and smoking induce oxidative stress. That oxidative stress subsequently damages the BBB through mitochondrial dysfunction and ROS production, followed by excitotoxicity, altered iron metabolism, inflammatory responses, pyroptosis, and necroptosis in the neurovascular unit [263]. Inflammation is also associated with BBB dysregulation through pathways mediated by inflammatory cytokines and lipid inflammatory mediators. In severe inflammation, parenchymal infiltration of peripheral immune cells and activation of MMPs lead to BBB structural damage [264]. Accumulating evidence demonstrates that *APOE*-ε4 leads to BBB dysfunction [265] where the mechanism is likely mediated by excess activation of cyclophilin A-MMP9 pathway in pericytes [266].

#### ISF/CSF drainage disturbances

Diffusion tensor imaging-based analysis along the perivascular space (DTI-ALPS) index has been used to evaluate the glymphatic clearance function [267]. The ALPS index is associated with cSVD neuroimaging markers including WMHs, lacunas, microbleeds, and enlarged perivascular spaces [268]. However, the ALPS index is correlated with cognitive function independent of other factors [269]. Furthermore, lower ALPS index is associated with lower Aβ42 levels in CSF [270]. Glymphatic impairment is predicted to stagnate ISF/CSF drainage and exacerbate brain accumulations of deleterious protein/cell debris, which eventually leads to cSVD-related cognitive impairment. IPAD disturbance also contributes to perivascular space enlargement in the white matter and CAA formation [271]. Thus, ISF/CSF drainage dysregulation through the glymphatic system and IPAD pathways should also be a key etiological mechanism of cSVD. Although CAA is mainly detected between smooth muscle cell layers of leptomeningeal arteries and penetrating arterioles in the IPAD pathway [272], Aβ deposition is sometimes detected in capillaries, venules, and veins [273]. While arterial CAA is fivefold more frequent compared to venous CAA in AD cases [274], Aβ deposition in veins are observed in 78% of severe CAA cases with cerebral hemorrhage [275]. An animal study using a TgF344-AD rat model showed that arteriolar Aβ accumulation precedes venular Aβ accumulation [276]. These observations suggest that IPAD and glymphatic dysfunction are connected. Continuous IPAD dysfunction causes arterial CAA and subsequentially compromises the glymphatic pathway resulting in Aβ deposition on venous vessels in severe cases. Cerebrovascular pulsatility has been identified as the driving force of ISF/CSF bulk flow along cerebral vessels. Thus, altered vascular wall compliance and reactivity due to cerebrovascular damages might disturb the homeostasis of IPAD and glymphatic system in cSVD [277]. Ultrafast magnetic resonance encephalography (MREG) shows that cardiac pulsations drive fluid drainage along periarterial spaces, whereas respiratory pulsations mediate perivenous fluid flow [278]. Hence, altered cardiovascular or respiratory systems also impact the IPAD and glymphatic drainage pathways. In addition, astrocytic AQP4 plays an essential role in regulating brain water homeostasis and glymphatic clearance system [88]; mislocalization or reduction of AQP4 is detected in white matter with cSVD [279].

#### Vascular inflammation

Vascular inflammation is often characterized by increases of homocysteine, ICAM-1 (intercellular adhesion molecule 1), VCAM-1 (vascular cell adhesion molecule 1), lipoprotein-associated phospholipase A2 (Lp-PLA2), VEGF, E-selectin, P-selectin, MMP9, neopterin, or CD40 [280]. Vascular inflammation is causatively or consequently involved in oxidative stress, vascular endothelial dysfunction [281], BBB damage [282], atherosclerotic plaque formation [283], narrowing of the lumen [284], and hemodynamic impairment [285], all of which eventually culminate in the development of cSVD (Fig. 3). A meta-analysis indicated that vascular inflammation associates with cSVD development in the brain regions supplied by deep perforating arteries such as basal ganglia [280]. The increase of macrophage-derived proinflammatory enzyme Lp-PLA2 has been shown as an risk factor for WMHs [285] as well as cardiovascular disease and stroke [286]. In addition, higher baseline levels of systemic inflammatory markers likely predict cSVD severity and progression [280]. Neutrophil count is also suggestively correlated with increased cSVD burden and prevalence [139]. Animal studies have demonstrated robust association between inflammation and cSVD, providing strong evidence that inflammation could be a major etiological factor of cSVD [287, 288]. Indeed, cSVD risk factors such as aging [289, 290] and hypertension [291, 292] have been known to cause both systemic and vascular inflammation.

### Preventative and therapeutic strategy for cSVD

Since cSVD is often a secondary phenotype of a metabolic syndrome, pharmacological approaches to ameliorate hypertension and atherosclerosis are the current standard to treat cSVD [293]. A meta-analysis reported that patients treated with intensive anti-hypertensive drugs have significantly slower WMH progression compared with non-treated groups [294]. While statins are 3-hydroxy-3-methylglutaryl coenzyme A (HMG-CoA) reductase inhibitors used to treat hyperlipidemia, a randomized controlled trial showed that a low dose of rosuvastatin treatment over 5 years suppresses WMH progression and reduces the risk for microbleeds in aged hypertension patients [295]. Pre-stroke statin medication also slowed post-stroke WMH progression during 2-year follow-up [296]. However, another study found that statins may exacerbate CAA-related lobar hemorrhage risk in *APOE*-ε4/ε4 and *APOE*-ε2/ε4 carriers [297]. Other reports failed to detect preventive effects of anti-hypertensive drugs and statins on WMH progression [298–301].

Antiplatelet therapies using aspirin, clopidogrel, dipyridamole, cilostazol, and ticagrelor are a main strategy for secondary stroke prevention as platelet activation predominantly causes vessel occlusion [302]. Antiplatelet therapy in secondary stroke prevention after lacunar stroke has been reported to reduce recurrence of any stroke and ischemic stroke with superior effects in dual-antiplatelet therapy compared to single-antiplatelet therapy [303]. However, long-term dual antiplatelet therapy with clopidogrel and aspirin resulted in increased rate of major bleeding and all-cause mortality than aspirin alone [304]. Prolonged antiplatelet therapy needs careful consideration when used for cSVD patients especially with CAA. As cilostazol administration showed the lower incidence of hemorrhagic stroke in lacunar stroke patients than aspirin, cilostazol appears to be safer treatment for cSVD [305]. Cilostazol has also been known to ameliorate cognitive decline and gliovascular damage through endothelial stabilization [306].

Although aducanumab and lecanemab have been approved by FDA to treat patients with early AD by reducing brain Aβ amyloids, it is not recommended for cases with diagnosed CAA [307]. Amyloid-related imaging abnormalities (ARIA) were reported in significant number of patients receiving anti-amyloid immunotherapy for AD, in particular in individuals carrying *APOE4* gene allele in a gene dose-dependent manner [308, 309]. CAA is likely involved in the pathogenic mechanism of ARIA as *APOE4* also increases the prevalence of CAA [310]. ARIA-E is characterized by vasogenic parenchymal edema or leptomeninges/sulci sulcal effusions. ARIA-H exhibits microhemorrhages or superficial siderosis hemosiderin deposits. Antibodies used in Aβ immunotherapy may directly attack vascular amyloid deposition, causing ARIA. Aβ-targeted immunotherapy could also increase perivascular Aβ accumulation, further exacerbating CAA and ARIA. ARIA is generally managed through temporary treatment suspension or dosage reduction, pulsed steroid therapy may ameliorate it [311].

Behavioral metrics (smoking, BMI, physical activity, and diet) are associated with WMH and lacunes risk [312]. Thus, lifestyle interventions are promising approaches for cSVD therapy. While smoking cessation is essential in current smokers with cSVD, multidomain intervention (diet, exercise, cognitive training, vascular risk monitoring) could improve or maintain cognitive functioning in the elderly [313]. Since exercise [314] and the Mediterranean diet improve endothelial function. [315], they could be beneficial in treating cSVD patients. Furthermore, the association between low serum vitamin B_12_ levels and increased white matter volume was identified [316]. A study reported that B-vitamin (folate, vitamins B_12_ and B_6_) supplementation lowers plasma homocysteine levels and reduces WMH burden in patients with severe cSVD intervention [317]. Vitamin E tocotrienols were also found to be beneficial in the attenuation of WMHs among cognitively unimpaired individuals [318].

There are also several emerging new therapeutic strategies for cSVD based on animal studies. For example, an angiotensin II receptor blocker, candesartan, attenuates vascular distensibility and cerebral blood flow by modulating pathological extracellular matrix accumulation in CARASIL model mice [319]. In addition, several active or passive immunotherapy strategies targeting NOTCH3 have been reported to be effective for CADASIL in mouse models [320–322]. Since nicotinamide mononucleotide supplementation could restore cerebrovascular endothelial dysfunction and improve cognitive function in aged wild-type mice, nicotinamide mononucleotide may pose protective effects age-related VCID [323]. Furthermore, G-CSF administration has been shown to restore white matter damage and improve non-spatial cognitive function in spontaneously hypertensive rats [324]. Minocycline could also reduce white matter damage, improved behavior, and prolonged life in spontaneously hypertensive/stroke prone rats [325].

## Conclusions

As contributions of VCID to age-related cognitive decline have been increasingly recognized, greater understanding of cSVD pathophysiology and etiology is desired to develop novel diagnostic and therapeutic strategies for the disease. Given that hypertension, smoking, and diabetes are strong risk factors for cSVD, it is reasonable to consider cSVD as a primary phenotype of metabolic syndrome in the brain. Although how these risk factors relate to each aspect of cSVD pathogenesis remains to be elucidated, lifestyle interventions focusing on vascular health might be the most effective approach to reduce cSVD risk, cardiovascular diseases, and stroke. In most of cSVD cases, clinical symptoms silently progress for many years before symptoms become evident. Although further studies are needed to define the adequate therapeutic window, earlier intervention at the pre-symptomatic stage should be beneficial to treat cSVD. Current advancements in neuroimaging enables precise cSVD diagnosis. However, there is a difficulty in predicting the onset and progression of cSVD. Early accurate diagnosis or prediction of cSVD may be accelerated through easily identifiable fluid biomarkers. Accumulating evidence indicates etiological pathways such as hypoperfusion/hypoxia, BBB dysregulation, ISF/CSF drainage disturbances, and vascular inflammation in cSVD, where a variety of cell types are involved in the pathogenesis. Since these different aspects of cerebrovascular damages are likely associated with one another, it might be critical to target multiple pathways to establish effective cSVD therapies.

In addition, cSVD might be causatively and consequently involved in AD pathogenesis as CAA is a common type of cSVD. While cSVD and AD frequently coexist in the elderly, future studies should further define how ameliorating cSVD phenotypes influence the onset and development of AD.

## Data Availability

Not applicable.

## Abbreviations

VCID: Vascular cognitive impairment and dementia
AD: Alzheimer’s disease
CSVD: Cerebral small vessel disease
ACA: Anterior cerebral artery
MCA: Middle cerebral artery
PCA: Posterior cerebral arteries
CSF: Cerebrospinal fluid
ISF: Interstitial fluid
IPAD: Intramural periarterial drainage
BBB: Blood–brain barrier
VEGF: Vascular endothelial growth factor
ZO-1: Zonula occludens-1
VE-cadherin: Vascular endothelial-cadherin
PECAM-1: Platelet endothelial cell adhesion molecule-1
Neural (N)-cadherin: N-cadherin
ABC: ATP binding cassette
IP3R: Inositol 1, 4, 5-trisphosphate receptor
NO: Nitric oxide
EET: Epoxyeicosatrienoic acids
ATP: Adenosine triphosphate
PLA2: Phospholipase A2
mGluR: Metabotropic glutamate receptor
PGE_2_: Prostaglandin E2
NMDA: N-methyl-D-aspartate receptor
AMPA: α-Amino-3-hydroxy-5-methyl-4-isoxazolepropionic acid receptor
nNOS: Neuronal NO synthase
COX: Cyclooxygenase
cGMP: Cyclic guanosine monophosphate
PKG: Protein kinase G
20-HETE: 20-Hydroxyeicosatetraenoic acid
PGI_2_: Prostaglandin I2
cAMP: Cyclic adenosine monophosphate
PKA: Protein kinase A
NPY: Neuropeptide Y
SON: Supraoptic nucleus
VP: Vasopressin
AT1: Angiotensin II type 1
eNOS: Endothelial NO synthase
ET: Endothelin
ONOO-: Peroxynitrite
NOX: NADPH oxidase
PDGF: Platelet-derived growth factor
PDGFRβ: PDGF receptor-β
VEGFR2: Vascular endothelial growth factor receptor-2
TGF-β: Transforming growth factor-β
TGFβR2: TGF-β receptor-2
Ang: Angiopoietin
MFSD2A: Major facilitator superfamily domain-containing 2a
LAMs: Leukocyte adhesion molecules
ANG-1: Angiopoietin-1
SHH: Sonic hedgehog
GDNF: Glial-derived neurotrophic factor
RA: Retinoic acid
IGF-1: Insulin-like growth factor-1
APOE: Apolipoprotein E
AQP4: Aquaporin 4
CAA: Cerebral amyloid angiopathy
Aβ: Amyloid-β
MRI: Magnetic resonance imaging
WMH: White mater hyperintensity
TFNEs: Transient focal neurological episodes
CADASIL: Cerebral autosomal dominant arteriopathy with subcortical infarcts and leukoencephalopathy
FLAIR: Fluid attenuation inversion recovery
GOM: Granular osmiophilic material
CARASIL: Cerebral autosomal recessive arteriopathy with subcortical infarcts and leukoencephalopathy
HTRA1: HtrA serine peptidase/protease 1
HDLS: Hereditary diffuse leukoencephalopathy with spheroids
MELAS: Mitochondrial encephalomyopathy, lactic acidosis, and stroke-like episodes
α-Gal-A: Alpha-galactosidase A
CRP: C-reactive protein
IL-6: Interleukin 6
HIF: Hypoxia-inducible factor
BMI: Body mass index
ROS: Reactive oxygen species
RAGE: Receptors for advanced glycation endproducts
MMP: Matrix metalloproteinase
HDL: High-density lipoprotein
LDL: Low-density lipoprotein
NF-κB: Nuclear factor (NF)-κB
NEMO: NF-κB essential modulator
RIPK3: Receptor-interacting protein kinase 3
GWAS: Genome-wide association studies
DTI-ALPS: Diffusion tensor imaging-based analysis along the perivascular space
MREG: Magnetic resonance encephalography
ICAM-1: Intercellular adhesion molecule
VCAM-1: Vascular cell adhesion molecule
Lp-PLA2: Lipoprotein-associated phospholipase A2
HMG-CoA: 3-hydroxy-3-methylglutaryl coenzyme A
ARIA: Amyloid-related imaging abnormalities
